# Maternal and neonatal outcomes following magnesium sulfate in the setting of chorioamnionitis: a meta-analysis

**DOI:** 10.1007/s00404-023-07221-3

**Published:** 2023-09-28

**Authors:** Vasilios Pergialiotis, Ioakim Sapantzoglou, Kalliopi Rodolaki, Antonia Varthaliti, Marianna Theodora, Panagiotis Antsaklis, Nikolaos Thomakos, Sofoklis Stavros, Georgios Daskalakis, Aggeliki Papapanagiotou

**Affiliations:** 1 First Department of Obstetrics and Gynecology, “Alexandra” General Hospital, National and Kapodistrian University of Athens, Athens, Greece; 2 Department of Biological Chemistry, Medical School, National and Kapodistrian University of Athens, Athens, Greece; 3 Third Department of Obstetrics and Gynecology, Attikon General Hospital, National and Kapodistrian University of Athens, 2, Lourou Str., 11523 Athens, Greece

**Keywords:** Magnesium sulfate, MgSO_4_, Chorioamnionitis, Neonatal outcome, Meta-analysis

## Abstract

**Purpose:**

Magnesium sulfate (MgSO_4_) has been widely used in obstetrics as a mean to help decrease maternal and neonatal morbidity in various antenatal pathology. As a factor, it seems to regulate immunity and can, thus, predispose to infectious morbidity. To date, it remains unknown if its administration can increase the risk of chorioamnionitis. In the present meta-analysis, we sought to accumulate the available evidence.

**Methods:**

We systematically searched Medline, Scopus, Clinicaltrials.gov, EMBASE, Cochrane Central Register of Controlled Trials CENTRAL, and Google Scholar databases in our primary search along with the reference lists of electronically retrieved full-text papers.

**Results:**

Eight studies were included that investigated the incidence of chorioamnionitis among parturient that received MgSO_4_ and control patients. Magnesium sulfate was administered in 3229 women and 3330 women served as controls as they did not receive MgSO_4_. The meta-analysis of data revealed that there was no association between the administration of magnesium sulfate and the incidence of chorioamnionitis (OR 0.98, 95% CI 0.73, 1.32). Rucker’s analysis revealed that small studies did not significantly influence the statistical significance of this finding (OR 1.12, 95% CI 0.82, 1.53). Trial sequential analysis revealed that the required number to safely interpret the primary outcome was not reached. Two studies evaluated the impact of MgSO_4_ in neonates delivered in the setting of chorioamnionitis. Neither of these indicated the presence of a beneficial effect in neonatal morbidity, including the risk of cerebral palsy, intraventricular hemorrhage, necrotizing enterocolitis, bronchopulmonary dysplasia, sepsis, stillbirth, or neonatal death.

**Conclusion:**

Current evidence indicates that magnesium sulfate is not associated with an increased risk of maternal chorioamnionitis. However, it should be noted that its effect on neonatal outcomes of offspring born in the setting of chorioamnionitis might be subtle if any, although the available evidence is very limited.

## Introduction

Magnesium is an essential metal in human physiology, belonging among the most important cations following potassium, calcium, and sodium, being the second most important cation in the intracellular environment [1]. As an intracellular component, its presence is essential for the processes that involve the formation and structure of the cellular membrane, the ribosomes, and the nucleus. Specifically, it has been associated with the regulation of mitochondrial activity, the activity of more than 500 enzymes, the cleavage of mRNA, the modulation of catabolic processes, and the neuromuscular activity [2, 3]. It is estimated that an average adult contains approximately 24 g of magnesium and its homeostasis is regulated by the kidneys and the bowel [4]. Hypomagnesemia is observed when the levels of magnesium fall below the limit of 1.8 mg/dl and is observed in approximately 15% of the adult population.

During pregnancy, magnesium has been administered as a treatment of several antenatal pathological entities. In the form of magnesium sulfate, it has been used to prevent eclampsia in preeclamptic patients and it has been also considered as a tocolytic agent [5–7]; however, given the limited amount of evidence and the lack of an evidential association between its use and actual clinical benefit for these two pathological entities, current recommendations do not include it as a treatment option. Currently, the use of magnesium sulfate is strongly recommended in cases with anticipated preterm birth as an offspring neuroprotective regimen [8, 9]. It is associated with a significant reduction of cerebral palsy and substantial gross motor dysfunction at 2 years of age [10].

To date, it remains unclear if magnesium sulfate intake during pregnancy may result in adverse maternal and neonatal outcomes; however, recent meta-analyses suggest that there is absence of evidence from randomized trials and that the risk of neonatal death or other, rarer adverse events as well as the risk of severe postpartum hemorrhage is rather small and does not differ compared to that of women that do not receive magnesium sulfate [11, 12].

Chorioamnionitis is a relatively rare antenatal entity that is encountered in approximately 9.7 per 1000 live births [13]. It is a significant factor that is independently associated with offspring mortality and the use of antibiotics is strongly supported as the anticipated reduction of neonatal death reaches 30% [13]. Magnesium is a significant component that modulates immunity as it seems that its levels are directly related to immunometabolism [14] and can therefore regulate the incidence as well as the outcome of pregnancies complicated by infectious diseases, including chorioamnionitis. Fetuses exposed to antenatal magnesium sulfate express reduced cytokine production and this effect is thought to be the principal factors that helps prevent cerebral palsy [15].

Considering these, we sought to investigate the potential effect of magnesium sulfate on the incidence of chorioamnionitis in pregnant women that receive antenatal treatment, irrespective of the underlying cause. Together, we opted to gather information relevant to the incidence of adverse maternal and neonatal events in pregnant women with chorioamnionitis that receive magnesium sulfate and compare them to patients with chorioamnionitis that do not receive magnesium sulfate.

## Methods

The present systematic review was registered in PROSPERO (International prospective register of systematic reviews) prior to its onset (Registration number: CRD42023398579) and is designed according to the Preferred Reporting Items for Systematic Reviews and Meta-Analyses (PRISMA) guidelines [16]. The review is based on aggregated data that have been already published in the international literature. Patient consent and institutional review board approval were, therefore, waived.

### Eligibility criteria, information sources, and search strategy

The eligibility criteria for the inclusion of studies were predetermined. Two correlations between magnesium sulfate and chorioamnionitis were presumed and investigated. The first regarded the incidence of chorioamnionitis among women that received magnesium sulfate and those that did not. The second aimed to evaluate the incidence of maternal and neonatal adverse events among women with chorioamnionitis that received and did not receive magnesium sulfate. The reason for administering magnesium sulfate in pregnancy was anticipated to vary among included studies and includes, but is not necessarily limited to, neonatal neuroprotection, prevention of preeclampsia, and prevention of preterm birth. Due to the anticipated limited number of studies, we selected to include all cases and perform subgroup analyses for the different groups, if possible. Case reports, experimental studies, and conference proceedings were excluded from the present meta-analysis.

**Fig. 1:**
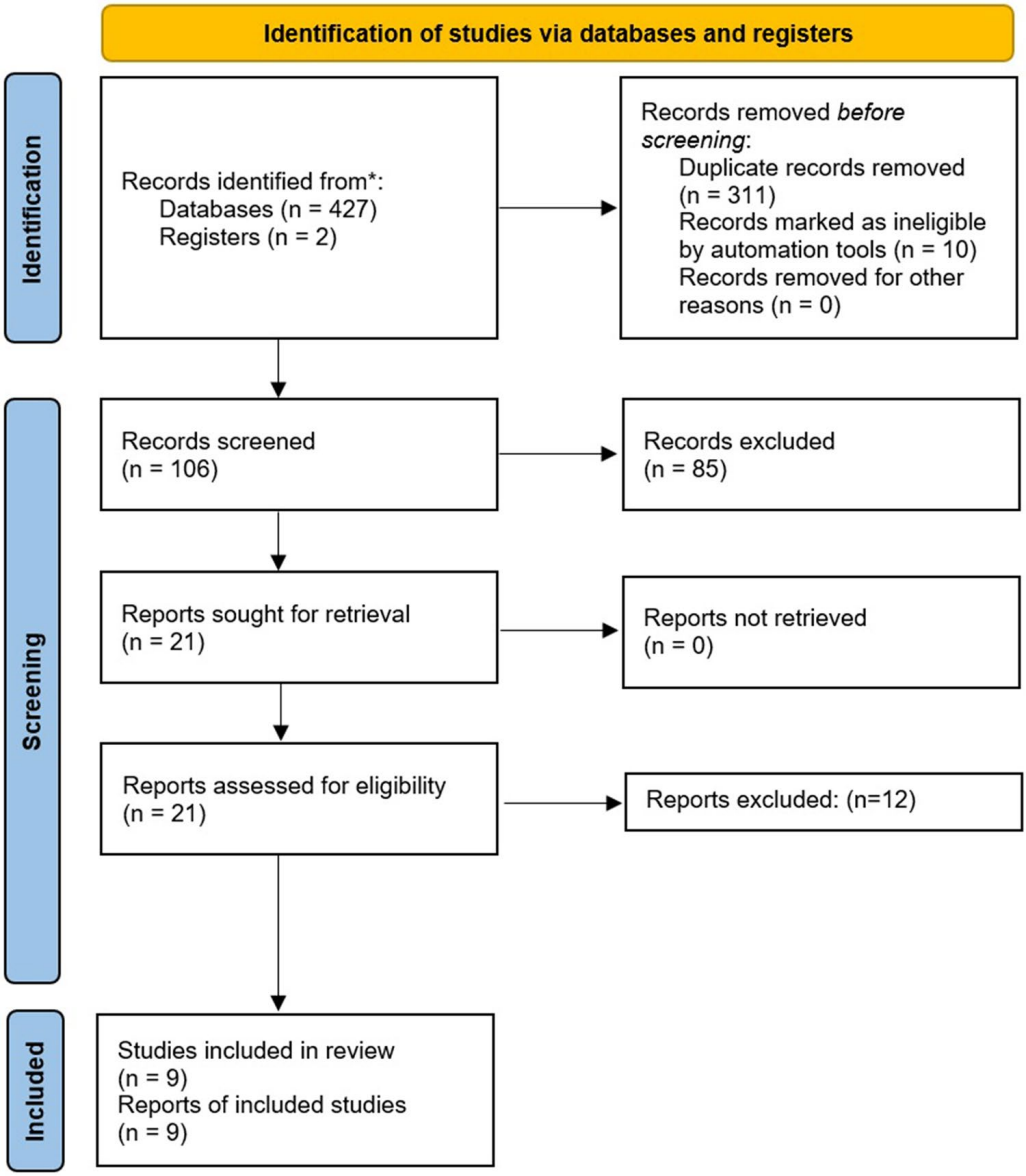
Search plot diagram

We used the Medline (1966–2023), Scopus (2004–2023), Clinicaltrials.gov (2008–2023), Cochrane Central Register of Controlled Trials CENTRAL (1999–2023), and Google Scholar (2004–2023) databases in our primary search along with the reference lists of electronically retrieved full-text papers. The date of our last search was set at April 30 2023. Our search strategy included the text words “magnesium sulfate; chorioamnionitis; endometritis” and is briefly presented in Fig. 1.

### Study selection

The process of study selection involved three consecutive stages. First, deduplication of retrieved articles was performed, and afterward, the titles and abstracts of all electronic articles were screened by two authors to evaluate if they provided relevant data. Studies were selected for inclusion following retrieval and review of the full text. Discrepancies that arose in this latter stage were resolved by consensus from all authors.

### Data extraction

Outcome measures were predefined during the design of the present systematic review. Data extraction was performed using a modified data form that was based on Cochrane’s data collection form for intervention reviews for RCTs and non-RCTs considering the anticipated maternal and neonatal adverse outcomes in cases with chorioamnionitis [17]. The primary outcome of our study was to evaluate differences in the incidence of chorioamnionitis among pregnant women that received magnesium sulfate during pregnancy and those that did not. Secondary outcomes included differences in inflammatory biomarkers (white blood cells, c-reactive protein levels), differences in the incidence of neonatal sepsis, neonatal respiratory distress syndrome, neonatal death, maternal endometritis, postpartum hemorrhage, and maternal sepsis.

### Assessment of risk of bias

The methodological quality of included randomized controlled trials was initially designed to be assessed by two independent reviewers using the risk of bias 2 (RoB 2) tool. The quality of non-randomized trials was assessed with Risk of Bias in non-Randomized Trials (ROBINS-I) tool which incorporates 7 domains that investigated bias that arises (i) from confounders, (ii) from selection of participants, (iii) from selective reporting in intervention measures, (iv) from deviations from intended interventions, (v) due to missing data, (vi) from selective reporting in outcome measures, and (vii) from selective reporting of outcomes.

### Data synthesis

Statistical meta-analysis was performed with RStudio using the *meta* function [RStudio Team (2015). RStudio: Integrated Development for R. RStudio, Inc., Boston, MA URL http://www.rstudio.com/]. Statistical heterogeneity was not considered during the evaluation of the appropriate model (fixed effects or random effects) of statistical analysis as the considerable methodological heterogeneity (Table 1) did not permit the assumption of comparable effect sizes among studies included in the meta-analysis [18]. Confidence intervals were set at 95%. We calculated pooled risk ratios (OR) and 95% confidence intervals (CI) with the Hartung–Knapp–Sidik–Jonkman instead of the traditional Dersimonian–Laird random-effects model analysis (REM). The decision to proceed with this type of analysis was taken after considering recent reports that support its superiority compared to the Dersimonian–Laird model when comparing studies of varying sample sizes and between-study heterogeneity [19]. Publication bias was not assessed due to the small number of included studies [20].

**Table 1:** Methodological characteristics of included studies

Year; author	Study type	Inclusion criteria	Exclusion criteria	Primary outcome	Indication for MgSO_4_
2003; Livingston	RCT	Singleton or twin pregnancies/both term and preterm or in postpartum period/Development of mild PE prior to labor/Development of mild PE postpartum/Admission for planned CS with mild PE	Chronic hypertension/Severe PE	Severe PE/eclampsia	Prevention of eclampsia
2008; Rouse	RCT	Singleton or twin pregnancies /24–31 + 6 weeks of gestational age/High risk for spontaneous delivery/Anticipation of an indicated preterm delivery within 2 to 24 h	Delivery anticipated within less than 2 h/Cervical dilatation exceeded 8 cm/Rupture of the membranes before 22 weeks/Major fetal anomalies or death/Maternal hypertension or PE/Receipt of intravenous magnesium sulfate within the previous 12 h	Moderate or severe cerebral palsy at or beyond 2 years of corrected age	Fetal neuroprotection
2015; Kamyar	RCT	Singleton pregnancies/24–31 + 6 weeks of gestational age/High risk for spontaneous delivery/Non anomalous pregnancies/Women with clinical chorioamnionitis	No chorioamnionitis/Twin pregnancies/Lacking delivery information/Consent not provided	Composite of stillbirth/death by the age of 1 year/Moderate or severe cerebral palsy at or beyond 2 years of corrected age	Fetal neuroprotection
2005; Deering	Retrospective cohort	Singleton or twin pregnancies/Preterm infants (≤ 36 weeks of gestational age)/Admission to NICU	Congenital anomalies/Admission at greater than 24 h of life/Hydrops fetalis	Neonatal Score for Neonatal Acute Physiology	Tocolysis
2010; Amin	Prospective cohort	Singleton or twin pregnancies/28–33 weeks of gestational age infants/Maternal placental histopathology performed	TORCH infections/Chromosomal disorders/Cranio-facial anomalies/Unstable condition/High frequency ventilator for a reliable ABR testing between 24–48 h after birth	Association of histologic chorioamnionitis with neurologic impairment in 28–33 weeks GA infants	Fetal neuroprotection
2002; Elimian	Retrospective cohort	Singleton or twin pregnancies/23–34 weeks of gestational age with intact membranes or PROM/Exposure to magnesium sulfate as a tocolytic agent	Neonates exposed to magnesium sulfate for seizure prophylaxis or maternal PE	Neonatal morbidity and mortality	Tocolysis
2017; Jung	Retrospective cohort	Singleton pregnancies/PPROM/23–31 + 6 weeks of gestational age	Intrauterine infection/Significant vaginal bleeding/Placental abruption/Cord prolapse/Non-reassuring fetal status/Cervical dilatation > 4 cm/Multifetal gestation/Pregnancy associated hypertension/Fetal anomalies /Intrauterine growth retardation	Maternal, neonatal and neurodevelopmental outcomes	Tocolysis
2017; Edwards	RCT	Singleton pregnancies/24–31 weeks of gestational age/High risk of preterm birth	Multiple gestation/Chromosomal abnormalities/Stillbirth/Congenital anomalies	Rate of Cerebral palsy at 2 years of age	Fetal neuroprotection
2014; Horton	RCT	Singleton pregnancies/24–31 + 6 weeks of gestational age/PPROM/No evidence of labor	Cervical dilatation > 4 cm/Twin pregnancies/Suspected chorioamnionitis/Previous administration of magnesium sulfate/Delivery within 1 h after randomization	Rates of delivery within 48 h and 7 days from randomization (latency)	Fetal neuroprotection

*CS* cesarean section, *NICU* neonatal intensive care unit, *RCT* randomized clinical trial, *PE* preeclampsia, *PPROM* preterm premature rupture of membranes, *TORCH* toxoplasma, rubella, cytomegalovirus, herpes

The potential presence of small-study effects was evaluated with Rücker’s Limit Meta-Analysis and the possibility of p-hacking with inspection of the results of the p-curve analysis.

### Prediction intervals

Prediction intervals (PI) were also calculated, using the *meta* function in RStudio, to evaluate the estimated effect that is expected to be seen by future studies in the field. The estimation of prediction intervals considers the inter-study variation of the results and expresses the existing heterogeneity at the same scale as the examined outcome.

### Trial sequential analysis

To evaluate the information size, we performed trial sequential analysis (TSA) which permits investigation of the type I error in the aggregated result of meta-analyses performed for primary outcomes that were predefined in the present meta-analysis. A minimum of 3 studies was considered as appropriate to perform the analysis. Repeated significance testing increases the risk of type I error in meta-analyses and TSA has the ability to re-adjust the desired significance level using the O` Brien–Flemming a-spending function. Therefore, during TSA, sequential interim analyses are performed that permit investigation of the impact of each study in the overall findings of the meta-analysis. The risk for type I errors was set at 5% and for type II errors at 20%. The TSA analysis was performed using the TSA v. 0.9.5.10 Beta software (http://www.ctu.dk/tsa/).

## Results

### Incidence of chorioamnionitis

Overall, 8 studies investigated the incidence of chorioamnionitis among parturient that received MgSO_4_ and control patients [21–27]. Of those, 5 had a randomized design and 4 were observational studies. Magnesium sulfate was administered in 3229 patients and 3330 women served as controls as they did not receive MgSO^4^. Patient characteristics are depicted in the Appendix and did not differ among the two groups. Significant differences were observed in the indications for the administration of MgSO_4_ with 5 studies including fetal neuroprotection as the primary indication, 3 studies including MgSO^4^ as a tocolytic regimen, and 1 study introducing MgSO^4^ as a measure against preeclampsia (Table 1). As a result, inclusion and exclusion criteria as well as investigated primary outcomes also differed among the studies included.

**Fig. 2:**
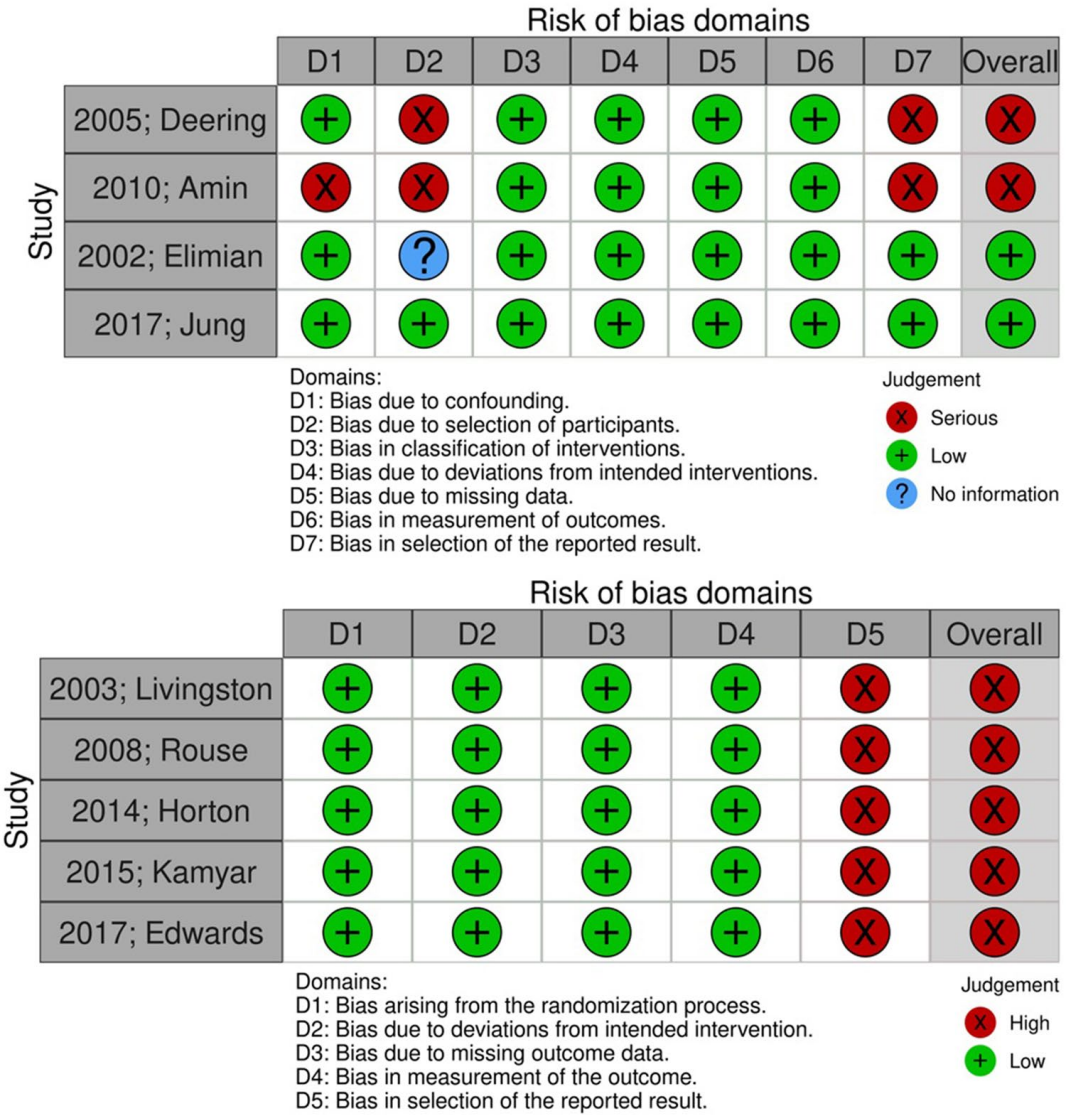
Upper section depicts the assessment of included non-Randomized Trials with the Risk of Bias (ROBINS-I) tool. Lower section depicts the assessment of included Randomized Trials with the Risk of Bias (RoB 2) tool

The methodological quality of included randomized trials revealed minor problems with selective reporting of clinical chorioamnionitis being the main issue encountered (Fig. 2). On the other hand, observational studies had more issues leaving apart the reporting of chorioamnionitis, which included concerns in the classification of interventions.

**Fig. 3:**
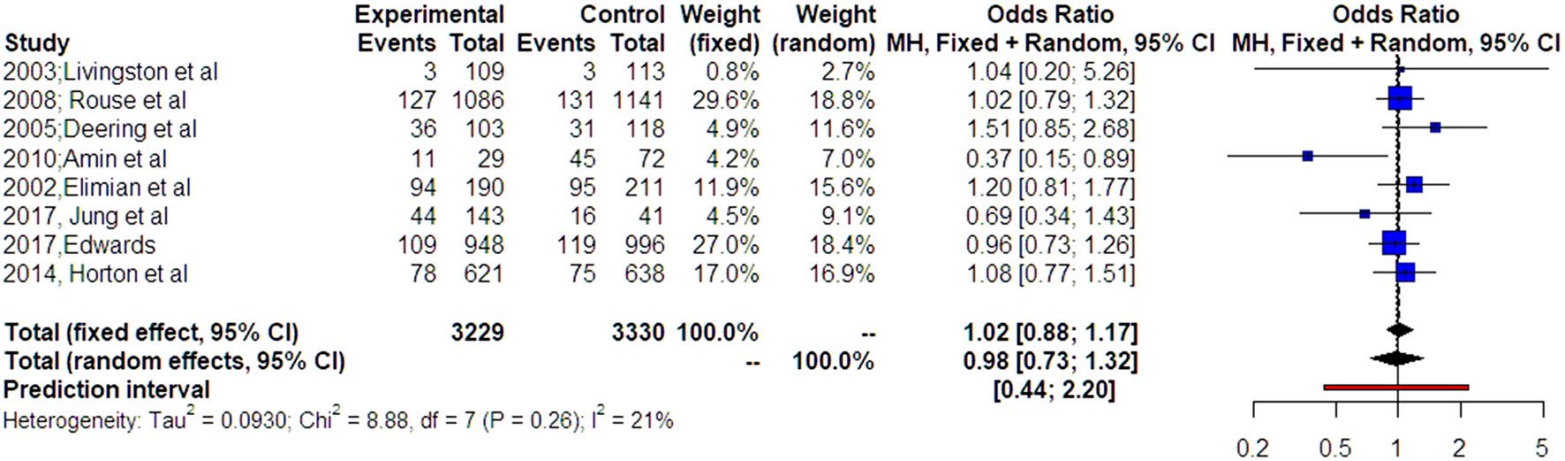
Odds ratio of chorioamnionitis among women receiving MgSO_4_ and controls. Forest plot analysis: Vertical line “no difference” point between the two groups. Red squares odds ratios, Diamond pooled odds ratios and 95% CI for all studies, Horizontal black lines 95% CI, Horizontal red line prediction intervals

The meta-analysis of data revealed that there was no association between the administration of magnesium sulfate and the incidence of chorioamnionitis (OR 0.98, 95% CI 0.73, 1.32) (Fig. 3). Rucker’s analysis revealed that small studies did not significantly influence the statistical significance of this finding (OR 1.12, 95% CI 0.82, 1.53). Moreover, there were no significant outliers detected; hence, the findings of individual studies should be considered homogeneous. As none of the included studies included significant results, analysis of p-hacking was not possible. Trial sequential analysis revealed that the required number to safely interpret the primary outcome was not reached.

### Neonatal adverse effects in neonates receiving MgSO_4_ in the presence of chorioamnionitis

Two studies evaluated the impact of MgSO4 in neonates delivered in the setting of chorioamnionitis [26, 28]. Neither of these indicated the presence of a beneficial effect in neonatal morbidity, including the risk of cerebral palsy, intraventricular hemorrhage, necrotizing enterocolitis, bronchopulmonary dysplasia, sepsis, stillbirth, or neonatal death. Of note, Edwards et al. observed that children receiving MgSO_4_ in the absence of chorioamnionitis had a significantly lower incidence of cerebral palsy and moderate/severe cerebral palsy at 2 years, indicating that the effect of the regimen might be more significant in the absence of intrauterine infection.

## Discussion

The findings of our study suggest that magnesium sulfate does not affect the risk of developing chorioamnionitis when administered antenatally as a mean to prevent maternal and neonatal pathology. Moreover, intake by pregnant women with chorioamnionitis does not help decrease the risk of neonatal death or the severe composite neonatal morbidity that is encountered during postpartum hospitalization. It should be noted, however, that these latter observations are based on limited data from two studies that involved a small number of parturient and neonates that does not suffice to consider them for practice recommendations. The actual neonatal benefit of MgSO4 in the presence of chorioamnionitis is questionable, although this observation might be attributed to the lack of adequately powered sample sizes.

The hypothetical linkage of MgSO_4_ and chorioamnionitis relies on evidence that seem to relate magnesium to the pathophysiology of immunomodulation in infectious diseases [29]. Magnesium is considered a fundamental ion for the human body that is strongly related to the innate and acquired immune responses [30]. Specifically, it has been linked to immunoglobulin synthesis, immune cell adherence, macrophage responses to cytokines, and T- and B- helper cells adherence to antigens. Experimental studies have shown that magnesium deprivation may increase proinflammatory proteins [31]. Similar findings have been shown in elderly patients that consume diets with low magnesium levels [32]. One could, therefore, speculate that magnesium intake might attenuate the severity of infectious diseases and this has been indeed proved in the clinical setting [30, 33, 34]. To date, unfortunately, in the field of obstetrics and particularly in cases with preterm premature rupture of the membranes, the beneficial effect of magnesium sulfate remains speculative as there is no evidence to support the pathophysiology behind this association.

The potential association between corticosteroid intake and chorioamnionitis should be considered as well. In the present systematic review, we observed significant discrepancies in the rates of corticosteroid administration among the studies that reported it with two of them noting a significant difference even among cases receiving MgSO4 and those that did not [22, 25]. One can assume, therefore, that this parameter may constitute a potential bias that deserves further attention in the future.

Concerning the neuroprotective effect of magnesium sulfate against cerebral palsy, it should be noted that one of the largest studies that was included in the present systematic review suggested that it is evident only among cases that do not develop chorioamnionitis (OR 0.52, CI 0.31–0.86), whereas its effect in cases with chorioamnionitis seems to be modest and rather insignificant (OR 0.76, CI 0.19–2.76) [26]. This particular difference should be taken into account when one considers the actual benefit of magnesium sulfate in preterm deliveries that are likely to result in neurodevelopmental deficiency and must be included in the patient counseling scheme. The actual linking mechanism between chorioamnionitis and offspring cerebral palsy has not been fully elucidated, and to date, the evidence linking these two entities seems to be particularly underpowered and biased [35]. Studies involving key factors that may provide evidence to support the correlation of chorioamnionitis with perinatal brain injury are still missing and most of the evidence dates more than 20 years ago speculating the development of a fetal inflammatory response syndrome that results in an inflammatory cascade that ultimately results in recruitment of immune cells and the release of growth factors in the developing brain [36]. The involved proteins disrupt the normal maturation of the central nervous system as well as the development of white matter and the effect is particularly aggravated in early preterm fetuses [37–40]. In the clinical setting, novel evidence suggests that intrauterine exposure to chorioamnionitis may provoke significant neuroanatomical alterations in the white matter, pallidum, and nucleus accumbens that can disrupt the physiology of the central nervous system [41]. These in turn result in cognitive dysfunction as white matter and the limbic system regulate behavior, motivation, long-term memory, as well as the ability to process novel information [42]. It remains unknown whether magnesium sulfate may help limit the side effects of chorioamnionitis or not; however, it is believed that magnesium intake, at least in adults, exerts a beneficial effect that limits neurodegeneration and cerebrovascular damage [43].

In this line, novel evidence focusing on neurogenic inflammation which is frequently associated with infection, toxin exposure, or traumatic brain injury [41] suggests that the process results in magnesium depletion which in turn triggers a cascade that results in secondary brain injury. Experimental studies involving rats have shown that Mg2+ deficient specimens have significantly larger cortical loss compared to animals with normal magnesium levels [44]. The neuroprotective effect of magnesium seems to be related to maintenance of the membrane potential as well as the capacity of cells to maintain their repair mechanisms [45]. Experimental studies in rodents suffering from meningitis have also implied the ability of magnesium therapy to improve the clinical outcome by attenuation of pneumolysin‐triggered pathogenic effects on the brain [46]. In humans, cerebrospinal fluid levels of magnesium seem to increase compared to serum levels in children with pyogenic and tubercular meningitis, indicating its detrimental function in the inflammatory process that targets the resolution of the disease [47].

### Implications for clinical practice and future research

Current evidence does not support the use of magnesium sulfate as a mean that may help reduce the risk of maternal chorioamnionitis, neither as an effective treatment that can limit the adverse neurodevelopmental effects that may be triggered in fetuses of patients with chorioamnionitis at risk of preterm delivery. It should be noted, however, that most of the data are drawn from retrospective series and the actual indications for the use of magnesium sulfate were particularly heterogeneous. In this line, we believe that future studies are needed to clarify if cases with histologic and/or clinical chorioamnionitis can be prevented in selected cases at risk of preterm delivery (particularly those with preterm premature rupture of the membranes) and if its use can help alleviate the associated neonatal adverse effects from the central nervous system. Predetermination of the actual groups of patients may be particularly useful as it is anticipated that the effect of magnesium may be more important in cases at risk of early preterm delivery as well as those with clinical signs of infection. Considering this information, we believe that it would be prudent to also determine the clinical factors that may be associated with increased risk of neonatal adverse effects in the presence of maternal chorioamnionitis and to closely monitor these patients. Recently, Brun et al. investigate the predictive accuracy of wearable sensors on the early detection of intraamniotic infection in the presence of preterm premature rupture of membranes [48]. Future studies might help evaluate if MgSO4 results in different fluctuations in these wearable sensors and whether it can actually determine the clinical course of patients with ruptured membranes.

## Conclusion

Magnesium sulfate intake does not correlate with chorioamnionitis in women treated for various antenatal pathologies. However, it remains unknown whether selected populations, including women with preterm premature rupture of the membranes, as well as neonates exposed to chorioamnionitis may benefit from its administration. Further studies are needed to guide clinical practice.

## Data Availability

Data will be available upon reasonable request.

## Appendix

See Table

**Table 2:** Patient characteristics (MgSO4 and incidence of chorioamnionitis)

Year; author	*N*	Age	BMI	Race (White/Black/Other)	DM	Nulliparous	Parity	Smoking	Corticosteroids	Antibiotics	Gestational age	PPROM	PPROM	CS
2003; Livingston	109 vs 113	22 vs 21.9	n/a	n/a	n/a	n/a	0.8 vs 0.9	n/a	n/a	n/a	38.4 vs 38.5	n/a	n/a	30 vs 27
2008; Rous	1096 vs 1145	26.1 vs 25.9	26 vs 26.4	404/483/209 vs 418/495/232	n/a	391 vs 414	n/a	299 vs 319	1062 vs 1116	n/a	28.3 vs 28.2	947 vs 995	25.2 vs 24.4	417 vs 448
2005; Deering	103 vs 118	n/a	n/a	n/a	n/a	n/a	n/a	n/a	43 vs 28	n/a	29.7 ± 3.2 vs 31.3 ± 2.9	77 vs 108	n/a	75 vs 73
2002; Elimian	190 vs 211	30 vs 31.4	n/a	n/a	n/a	n/a	n/a	n/a	182 vs 130	136 vs 95	28.2 vs 29.3	49 vs 92	n/a	n/a
2017; Jung	143 vs 41	32 vs 32.7	n/a	n/a	n/a	75 vs 7	n/a	n/a	114 vs 18	n/a	29.4 vs 28	143 vs 41	n/a	51 vs 18
2014; Horton	621 vs 638	26.8 vs 26.1	26.3 vs 26.6	238/275/16 vs 238/286/16	n/a	195 vs 213	n/a	186 vs 182	602 vs 622	n/a	28.5 vs 28.3	621 vs 638	25.3 vs 24.9	n/a

2.
